# Dynamic Metabolic Response to Adriamycin-Induced Senescence in Breast Cancer Cells

**DOI:** 10.3390/metabo8040095

**Published:** 2018-12-15

**Authors:** Rong You, Jin Dai, Ping Zhang, Gregory A. Barding, Daniel Raftery

**Affiliations:** 1 College of Life Sciences, South China Normal University, 55 Zhongshan Avenue West, Guangzhou 510631, China; 20021141@m.scnu.edu.cn; 2 Mitochondria and Metabolism Center, Department of Anesthesiology and Pain Medicine, University of Washington, 850 Republican Street, Seattle, WA 98109, USA; daij@uw.edu (J.D.); ping17028@swu.edu.cn (P.Z.); gabarding@cpp.edu (G.A.B.J.); 3 College of Plant Protection, Southwest University, 2 Tiansheng Road, Chongqing 400715, China; 4 Chemistry and Biochemistry Department, California State Polytechnic University, Pomona, CA 91768, USA; 5 Fred Hutchinson Cancer Research Center, 1100 Fairview Avenue N., Seattle, WA 98109, USA

**Keywords:** senescence MCF7, MDA-MB-231, metabolomics, isotope tracing analysis, gas chromatography–mass spectrometry (GC–MS)

## Abstract

Cellular senescence displays a heterogeneous set of phenotypes linked to tumor suppression; however, after drug treatment, senescence may also be involved in stable or recurrent cancer. Metabolic changes during senescence can provide detailed information on cellular status and may also have implications for the development of effective treatment strategies. The metabolic response to Adriamycin (ADR) treatment, which causes senescence as well as cell death, was obtained with the aid of metabolic profiling and isotope tracing in two human breast cancer cell lines, MCF7 and MDA-MB-231. After 5 days of ADR treatment, more than 60% of remaining, intact cells entered into a senescent state, characterized by enlarged and flattened morphology and positive blue staining using SA-β-gal. Metabolic trajectory analysis showed that the two cell lines’ responses were significantly different and were divided into two distinct stages. The metabolic shift from the first stage to the second was reflected by a partial recovery of the TCA cycle, as well as amino acid and lipid metabolisms. Isotope tracing analysis indicated that the higher level of glutamine metabolism helped maintain senescence. The results suggest that the dynamic changes during senescence indicate a multi-step process involving important metabolic pathways which might allow breast cancer cells to adapt to persistent ADR treatment, while the higher level of anapleurosis may be important for maintaining the senescent state. Ultimately, a better understanding of metabolic changes during senescence might provide targets for cancer therapy and tumor eradication.

## 1. Introduction

Cellular senescence was initially identified as cell cycle arrest from the limited replicative capacity in normal human diploid fibroblasts (HDFs); it was termed “replicative senescence” and associated with telomere shortening or dysfunction [1]. More recently, it has been recognized that a number of different stressors including oncogenic mutations, chemotherapeutic drugs and oxidative stress can cause telomere-independent cellular senescence, which is termed premature senescence [2]. Senescent cells display heterogeneous phenotypes including enlarged cell size, flattened cell morphology, an inability to synthesize DNA, formation of senescence-associated heterochromatin foci (SAHF) and expression of an endogenous senescence-associated β-galactosidase activity (SA-β-gal) [3]. Premature senescence in cancer therapy serves as an effective tumor-suppressor by preventing cancer cell proliferation or blocking the acquisition of tumor transformation [4]. However, the senescence-associated secretory phenotype during cancer therapy may influence tissue microenvironments, and even stimulate tumorgenesis and metastasis in vitro and in vivo [5,6]. Moreover, premature senescent cancer cells have the capability to escape growth arrest and re-enter the cell cycle, leading to tumor relapse [7,8]. Thus, the process of tumor cells undergoing cell senescence after drug treatment results in stable disease rather than regression of the tumor, which represents a non-optimal outcome and significant health risk in cancer therapy.

Cellular survival and growth require specific metabolic reprogramming to adapt to genetic or environmental stresses because of the need for metabolic pathways to continue to produce energy, precursors and substrates for macromolecular synthesis and/or cell signaling. One of the hallmarks in cancer biology is the higher levels of glucose uptake and glycolysis during tumor growth, commonly known as the Warburg effect, as well as the importance of glutamine as an anapleurotic substrate for the TCA cycle [9]. It has been noted that metabolic reprogramming might be exploited therapeutically for cancer therapy. With the aim of providing further insights into cancer biology and new targets for cancer therapy, metabolic phenotypes associated with cell senescence have been studied over the last few years. Higher utilization of glucose and higher ATP production were observed in therapy induced senescent (TIS)-competent lymphomas [10]. Consequently, TIS lymphomas were sensitive to blocking glucose utilization, which led to their selective eradication. The mitochondrial gatekeeper pyruvate dehydrogenase (PDH) was found to be a crucial mediator of oncogene-induced senescence (OIS) by BRAF^V600E^, which was accompanied by increased pyruvate oxidation and mitochondrial oxidative phosphorylation [11]. A lower level of deoxyribonucleotide triphosphate was also found in oncogene-induced senescence, caused by oncogene-induced repression of ribonucleotide reductase subunit M2 [12]. These studies revealed that senescent cells display metabolically active and context-dependent phenotypes. It was also suggested that understanding the metabolic changes during cell senescence may have implications for the development of new and effective strategies to treat cancer.

Although significant progress towards understanding the senescence-associated phenotype and its underlying molecular mechanisms have been made recently, a global metabolic view at the systems level is still lacking. Metabolomics provides a comprehensive characterization of the metabolic changes in biological systems that occur in response to different stimuli, and their interpretation in terms of metabolic pathway changes allows an understanding of the physiological variation [13,14]. Metabolomics has found widespread applications in cellular metabolism for elucidating perturbed cellular homeostasis, cell transformation and stem cell differentiation [15,16,17] and providing an understanding of the metabolic control of cell fate. In addition, stable isotope tracing has become an important and complementary tool in metabolomics, allowing one to track individual atoms and determine the fate of individual metabolites, through which metabolic network and flux changes can be obtained to facilitate the identification of altered pathways [18,19]. Here, we use gas chromatography–mass spectrometry (GC–MS) based global metabolomics and isotope tracer analysis to identify the metabolic changes during the progression of senescence in two breast cancer cell lines induced by Adriamycin (ADR) treatment to better understand how senescent cells can maintain their metabolic activity. 

## 2. Results

**Figure 1:**
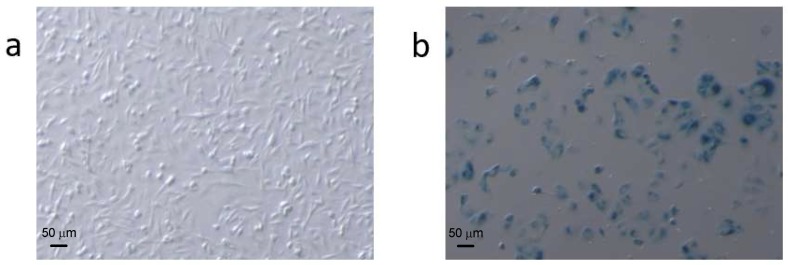
Comparison of (**a**) normal and (**b**) Adriamycin (ADR)-treated MCF7 cells stained for SA-β-gal activity. ADR-treated cells were larger and had a flatter morphology than untreated cells. Cell counting (250 cells per condition) showed that the number of SA-β-gal-positive (blue) cells versus the number of total cells was approximately 4% in the untreated cells and ~60% in the treated cells.

***Morphological characteristics of ADR-induced cell senescence***. ADR treatment enabled the preparation of stable and repeatable senescent cell samples in MCF7 and MDA-MB-231 breast cancer cell lines. ADR treated cells became flattened and enlarged (Figure 1), and the effects of ADR treatment on the breast cancer cells were dose and time dependent (data not shown). When the cells were treated with 0.04 μg/mL ADR for 5 days more than 60% of the remaining, adherent cells became senescent, according to SA-β-gal analysis, indicating that ADR treatment caused very significant cell senescence in MCF-7 and MDA 231 cells. Higher dosages of ADR in preliminary experiments results in significant cell death and thus were not useful to study senescence. By comparison, less than 4% of cells were senescent after 7 days of culture without ADR treatment.

**Figure 2:**
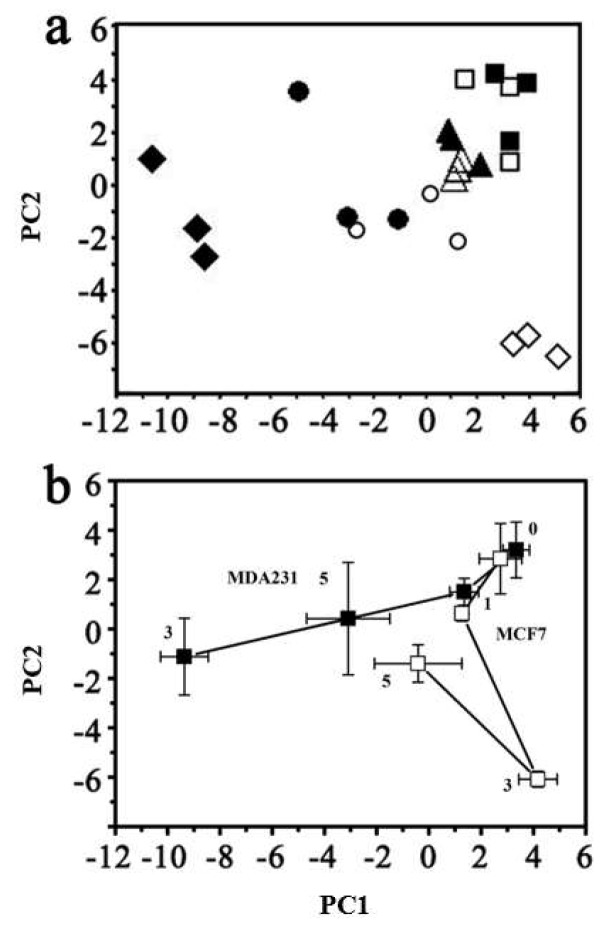
The overall metabolic response to ADR damage visualized using (**a**) principal component analysis (PCA) of all cell samples (R^2^ = 0.547 and Q^2^ = 0.367). MDA-MB-231 cells with ADR treatment for: ■ 0 days; ▲ 1 day; ◆ 3 days; ● 5 days. MCF7 cells with ADR treatment for: □ 0 days; △ 1 day; ◇ 3 days; ○ 5 days. (**b**) The centroided metabolic trajectories for the two cell lines during ADR treatment: ■ MDA-MB-231; □ MCF7. PC1 loading: 36.3%; PC2 loading: 18.4%.

***GC–MS based metabolomics characterization of cellular metabolic changes during ADR-induced senescence.*** Multivariate analysis of the global metabolic changes at 0, 1, 3, and 5 days of ADR treatment was performed using principal component analysis (PCA) (Figure 2) and showed large changes over the treatment time. The metabolic trajectories during ADR-induced cell senescence indicated that responses of these two cell lines to ADR-induced senescence were different. The two types of cell line samples were clustered together in the absence of ADR treatment. Distinct responses of MCF7 and MDA-MB-231 cells became evident after 3 days of ADR treatment (see Figure 2b), with the sample trajectory of MCF7 moving away from the initial pre-treatment cluster along PC2 while that of MDA-MB-231 moved away along PC1. However, after 5 days of ADR treatment, at a time when the two cell lines showed more than 60% cell senescence based on the SA-β-gal analysis, both cell lines moved partly back towards the initial clusters. 

Individual metabolites perturbed by ADR treatment were identified using the Student’s *t*-test. Since the 3-day ADR treatment caused the most obvious responses for both MCF7 and MDA-MB-231 as indicated by PCA, a direct comparison of each metabolite for the 3-day ADR treatment time point was performed with those samples prior to ADR treatment. A further comparison was carried out between the 3- and 5-day treatment periods to better understand the recovery trend. Metabolites with significantly altered levels are listed in Table 1 for MCF7 and Table 2 for MDA-MB-231. 

For MCF7, 2-keto-3-methylvaleric acid, an intermediate metabolite of branched-chain amino acid (BCAA) metabolism was elevated after ADR treatment. TCA cycle metabolites including fumaric acid, malic acid and citric acid decreased after 3 days of ADR treatment. However, these metabolites were significantly increased when MCF7 cells entered into senescence after 5 days of treatment. Meanwhile, amino acids associated with the TCA cycle, aspartic acid and glutamic acid, which are transformed from oxaloacetate and α-ketoglutarate, respectively, were also tracked with the TCA intermediates. The level of serine, which is transformed from intermediates in the glycolysis pathway was decreased after 3 days but recovered when cells entered into more than 60% senescence. Moreover, fatty acids in MCF7 including heptadecanoic acid, linoleic acid, oleic acid and stearic acid also initially decreased, followed by recovery from ADR-induced DNA damage after 5 days of ADR treatment. In all, the characteristics of these significantly changed amino acids, fatty acids and the intermediates of TCA cycles showed that ADR treatment caused obvious effects on MCF7 cell metabolism, most of which showed their lowest levels after 3 days of ADR treatment but which then later recovered after 5 days. 

For MDA-MB-231, changes in 2-keto-3-methylvaleric acid showed the same trend as for MCF7, which was elevated after ADR treatment. ADR treatment also caused obvious effects on the TCA cycle, lipid metabolism and amino acid metabolism (Table 2). The same downregulation of TCA intermediates, including fumaric acid, malic acid and citric acid occurred after 3 days of ADR treatment. However, a few amino acid and fatty acid changes in MDA-MB-231 cells were opposite to those observed in MCF7 cells. Specifically, alanine, glutamic and other amino acids were upregulated after 3 days to their highest levels, and then were reduced after 5 days of ADR treatment. These results suggest very different responses to ADR treatment based on cell type, even though both cell types showed the same morphological and SA-β-gal detected changes. 

**Figure 3:**
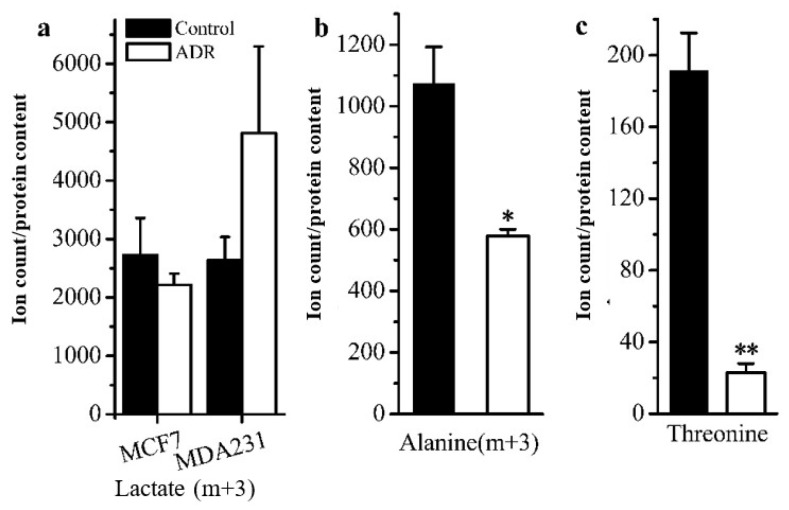
Comparison of cell metabolism between the ADR-treated senescent and non-treated cancer cells measured by isotope tracing analysis: (**a**) levels of the m + 3 isotopologue of lactate in MCF7 and MDA-MB-231; (**b**) levels of the m + 3 isotopologue of alanine in MCF7. (**c**) Measure of threonine levels in MCF7. * signifies *p* < 0.05 and ** signifies *p* < 0.005.

***GC–MS based isotope tracing analysis***. Stable isotope tracing analysis using [U-^13^C]-labeled glucose was also employed to compare the cellular metabolism of ADR treated and non-treated MDF7 and MDA-231 cancer cells to help identify how senescent cells maintain an active metabolic phenotype after 5 days of ADR treatment. From the results of tracing experiments, we observed that the levels of m + 3 isotopologues of lactate and pyruvate derived from labeled glucose in these two cell lines did not change in an obvious manner based on an analysis of the isotopologue distribution of metabolites (Figure 3a). However, the level of the m + 3 isotopologue of alanine in MCF7 was lower than that for non-senescent cells (Figure 3b), indicating that in senescent cells a lower flux of pyruvate to alanine occurred. Meanwhile, the m + 0 level of the essential amino acid threonine was lower in senescent MCF7 (Figure 3c), which might be related with higher threonine degradation (to α-ketoglutaric acid) in senescent MCF7 cells. 

**Figure 4:**
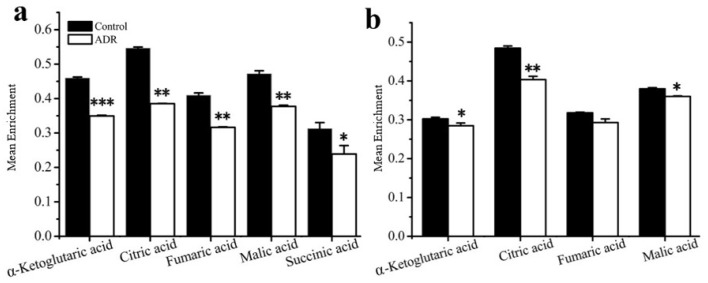
Mean enrichment of TCA cycle intermediates for non-treated cells and ADR-treated cells by isotope tracing analysis: (**a**) MCF7, (**b**) MDA-MB-231. * signifies *p* < 0.05; ** signifies *p* < 0.005 and *** signifies *p* < 0.0001.

**Figure 5:**
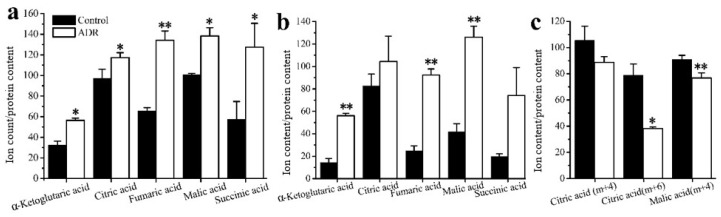
Distribution of the isotopologues of TCA cycle intermediates for non-treated cells and ADR-treated cells (**a**) (m + 2) levels of intermediates in MCF7 cells; (**b**) (m + 2) levels intermediates in MDA-MB-231 cells; (**c**) (m + 4) and (m + 6) isotopologues of citrate and malate. * signifies *p* < 0.05 and ** signifies *p* < 0.005.

**Figure 6:**
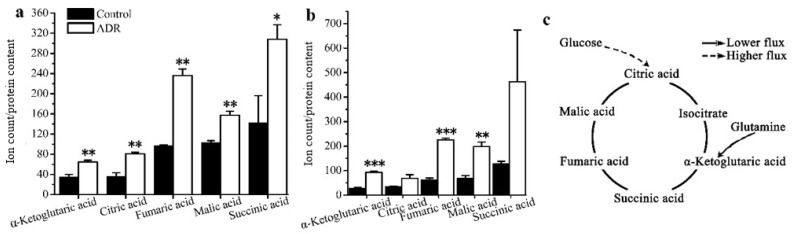
Levels of the (m + 0) isotopologue of TCA cycle intermediates for non-treated cells and ADR-treated cells: (**a**) MCF7; (**b**) MDA-MB-231. (**c**) A schematic characterization of TCA metabolites and how they might maintain cell senescence based on the isotopologue (m + 0) levels of TCA intermediates. * signifies *p* < 0.05; ** signifies *p* < 0.005 and *** signifies *p* < 0.0001.

The results also showed that the mean enrichments of TCA cycle intermediates derived from labeled glucose for senescent MCF7 and MDA131 were lower than those for the non-senescent cells; less glucose entered into the TCA cycle in senescent cells (Figure 4a,b). The doubly ^13^C-labeled isotopologues (m + 2) of citric, α-ketoglutaric, succinic, fumaric and malic acids in the TCA cycle significantly increased in the senescent cells (Figure 5a,b). However, lower levels of (m + 4), (m + 6) isotopologues of citric and malic acids appeared in senescent MCF7 (Figure 5c), which was also related to lower glucose entering the TCA cycle. It is also notable that a large fraction of these TCA metabolites (m + 0) were higher than those of the untreated cells (Figure 6a,b).

## 3. Discussion

DNA-damaging agents can induce premature senescence in cancer cells and, therefore, could provide an effective method to limit tumor progression by preventing cancer cell proliferation [20]. However, increasing evidence shows that prematurely senescent cells maintain their metabolic activity and have the ability to escape growth arrest to re-enter into the cell cycle [12,21]. Thus, tumor cells undergoing cell senescence after drug treatment results in stable disease rather than regression of the tumor and, therefore, represents a potential lurking hazard in cancer therapy that might lead to tumor relapse. In fact, the physiological consequences of senescent cancer cells still remain elusive. Only a few studies that address the potentially altered metabolism occurring under cell senescence have been performed [22,23]. In the recent work by Wu et al., senescence and apoptosis were contrasted using a combination of metabolomics and proteomics. The study revealed that senescent MCF7 cells underwent metabolic reprogramming to survive by facilitating reactive oxygen species (ROS) elimination and DNA damage repair, while the metabolism of apoptotic MCF7 cells was downregulated when faced with irreparable DNA damage. Nevertheless, the metabolic changes associated with the duration of stress-induced senescence are not sufficiently clear, nor how senescent cells maintain their metabolic activity. Understanding the metabolic processes important in cell senescence might have profound implications for the development of effective strategies to treat cancer and for finding related biomarkers to measure cellular responses.

The main focus in our study was to identify underlying metabolic alterations in ADR-induced breast cancer cell senescence and describe the metabolic reprogramming that occurs. With the aid of GC–MS based metabolomics and isotope tracing methods, we obtained detailed metabolic information associated with breast cancer cell senescence. Two cell human breast cell lines: MCF7 (p53 wild-type, estrogen receptor, ER+) and MDA-MB-231 (p53 mutant, estrogen receptor, ER−) were treated by moderate dosage of the anticancer drug ADR for 5 days to induce senescence, by which time more than 60% of the remaining, adherent breast cancer cells entered into cell senescence. Morphological characterization and staining analysis suggested that ADR-induced senescent cells represented reliable models for studying the metabolic events associated with response to chemotherapy-induced senescence. Dramatic metabolic changes in response to ADR-induced cell senescence were observed clearly using GC–MS based metabolomics. 

Metabolic responses were dependent on treatment time, indicating that these two cell lines might be very sensitive to the duration of ADR stress. Thus, the metabolic changes across multiple time points were needed to reflect changes during the progression of cell senescence. PCA analysis indicated that the two cell lines underwent different metabolic trajectories after ADR treatment. This result might be due in part to their different genetic backgrounds, which could contribute to the different metabolic phenotypes. In fact, cell senescence is not characterized by a stable and homogeneous status but rather a heterogeneous phenotype depending on cell types and diverse stimuli [24]. Moreover, one of the most striking features observed in our study was that the metabolic responses of MCF7 and MDA-MB-231 to ADR treatment were divided into different stages. For both cell lines, the metabolic profiles after 3 days of treatment were very different compared to their initial profiles. Unexpectedly, after 5 days of ADR treatment both cell lines exhibited some recovery as they entered into cell senescence. The dynamic nature and the two-stage development of the metabolic profiles might be correlated with a DNA damage response and metabolic adaption, respectively. Genotoxic stresses initiate related signaling pathways to repair DNA damage through the DNA damage response (DDR) machinery [25]. However, damaged cells can also be induced to undergo senescence with persistent DNA damage [26]. Ultimately, there may be a molecular switch that regulates cellular metabolic responses to genotoxic stresses. 

In MCF7 cells, the downregulation of TCA cycle metabolism was observed after the first stage. It has been reported that mitochondria are the primary target involved in ADR treatment [27], so the observed lowered metabolism may represent mitochondrial dysfunction and energy imbalance during the first stage. In addition, amino acid and fatty acid metabolism in MCF7 cells was also reduced during the first stage. The TCA cycle coordinates energy production and biosynthesis as well as the exit of intermediates such as malic, alpha-ketoglutaric and citric acids from the cycle to supply various biosynthetic pathways including amino acid and fatty acid synthesis. Thus, ADR treatment can induce decreases in TCA cycle metabolism concomitant with decreases in amino acid and fatty acid metabolism. 

An interesting finding in our study was the modulation of the concentrations of heptadecanoic acid in MCF7 cells. While heptadecanoic acid and other odd-chain fatty acids are normally considered of exogenous origin or produced from propionate, a recent study found that the cytidine-5-monophosphate/heptadecanoic acid metabolic ratio can be used as a powerful biomarker of breast cancer, implying that fatty acid synthesis is potentially regulated in breast cancer [28]. The regulation of heptadecanoic acid here might be related to fatty acid synthesis and membrane lipid metabolism changes. Even larger initial changes in heptadecaonic acid were observed for MDA-MB-231 cells.

The metabolic recovery during the second stage (day 3–5) was substantiated by the synchronous upregulation of TCA cycle, amino acid and fatty acid metabolisms as the MCF7 cells entered into senescence. We can conclude that in MCF7, TCA cycle metabolism is strongly coupled to ADR-induced mitochondrial changes during cell senescence. Previously, it was shown that iron accumulation in mitochondria occurs as a result of ADR and causes cardiotoxicity [29]. However, the metabolic response to DNA damage in MDA-MB-231 was more complicated and sometimes in contrast to changes observed in MCF7. Changes in the TCA cycle showed an unanticipated inverse relationship with amino acid and fatty acid metabolisms in the first stage of treatment. During the second stage, the recovery of citric acid levels, concomitant with the decrease in amino acid and fatty acid metabolisms also showed metabolic recovery in MDA-MB-231 after ADR treatment. However, the synchronization between TCA cycle metabolism and amino and fatty acids did not occur in MDA-MB-231 after ADR treatment. Instead, TCA cycle metabolism appears to be inversely correlated with amino acid and fatty acids metabolism. Our data suggest that MCF7 cells appear to have a higher level of basal respiration and an improved ability to sustain TCA cycle metabolite levels with loss of glucose oxidation after ADR treatment, while MDA-MB-231 cells were required to continue using amino acids as fuel, leading to their depletion and less anapleurotic recovery. 

Overall, the metabolic alteration of both MCF7 and MDA-MB-231 cells undergoing ADR-induced senescence passed through two different stages. The metabolic shift observed from the first stage to the second was likely caused by the partial adaptation of the cells to persistent ADR stress such that they could enter into senescence [30]. A number of signaling pathways would likely become activated in response to the metabolic imbalance during the periods of ADR treatment, including mTORC1, AMPK, and NFKB [31]. Therefore, such metabolic shifts might be explored as therapeutic targets to treat cancers to avoid senescence and possible relapse. In addition, we note that the level of 2-keto-3-methylvaleric acid, an intermediate of the BCAA catabolism pathway, increased for both MCF7 and MDA-MB-231. The observed change might be related to DNA protective effects, as higher levels of BCAAs have been suggested to elevate glutathione-S-transferase (GST) activities against DNA damage [32]. In addition, BCAAs can feed into the TCA cycle through the formation of acetyl-CoA or succinyl-CoA, and thus the elevation of 2-keto-3-methylvaleric acid might indicate the activation of a metabolic restorative mechanism. Considering ADR treatment caused obvious effects on TCA cycle metabolism, the higher level of BCAA metabolism also suggested a metabolic shift toward oxidative metabolism using non-glucose substrates (2-keto-3-methylvaleric acid) due to the inactivation of the TCA cycle.

Based on isotope-tracing analysis, both MCF7 and MDA-MB-231 exhibited almost the same metabolic characteristics after 5 days of ADR treatment. We observed that the glycolysis pathway in both ADR-induced senescent cell lines functioned similarly under senescence. There were no obvious distinctions in the levels of m + 3 isotopologues of lactate and pyruvate derived from labeled glucose between these two cell lines. This result suggests that the energy demands in senescent cells may not represent a prerequisite for maintaining the senescent status. Furthermore, the lower mean enrichment of TCA cycle metabolites in senescent MCF7 and MDA-MB-231 cells showed that the glucose flux into the TCA cycle was lower compared to pretreated cells, which might be related to mitochondrial damage and the activation of the tumor suppressor p53 upon ADR-induced cell senescence; p53 can down-regulate the expression of glucose transporters GLUT1 and GLUT4 [33]. Nevertheless, the isotopologue m + 2 levels of fumaric, malic, citric and alpha-ketoglutaric acids were increased, suggesting that flux through the first turn of the TCA cycle was higher in senescent cells. Since senescent cells appeared to develop a highly active secretory phenotype characterized by robust production of various inflammatory cytokines, the TCA cycle intermediates might need to exit the cycle to supply substrate for various biosynthetic pathways. If true, higher levels of m + 2 isotopologues might be related with the senescence associated secretory phenotype (SASP). The higher levels of m + 0 isotopologues of TCA cycle intermediates were observed in both senescent cell types, most of which could be supplied by glutamine anapleurosis or threonine in MCF7. Senescent cells may need a higher level of glutamine anapleurosis to survive DNA damage and maintain senescence. A number of studies have reported on glutamine’s ability to support the TCA cycle to produce energy and provide precursors for macromolecular synthesis for cell survival [34]. 

## 4. Materials and Methods

***Chemicals.*** MSTFA+1%TMCS (N,O-Bis(trimethylsilyl)trifluoroacetamide with 1% (vol/vol) Trimethylchlorosilane) (Thermo Fisher Scientific, Waltham, MA, USA), MTBSTFA (N-(*tert*-butyldimethylsilyl)-N-methyltrifluoroacetamide with 1% (*v/v*) (Sigma-Aldrich, St. Louis, MO, USA), and TBDMCS (*tert*-butyldimethylsilyl chloride) (Sigma-Aldrich) were purchased for metabolite derivatization. The FAME (fatty acid methyl-ester) library of compounds with different carbon chain lengths for retention index (RI) calculations was purchased from Agilent (Santa Clara, CA, USA). Methoxyamine hydrochloride (MeOX), chloroform, and pyridine were purchased from Sigma-Aldrich. Methanol (high-performance liquid chromatography (HPLC) grade) was purchased from Thermo Fisher Scientific. [U-^13^C] labeled glucose was purchased from Cambridge Isotope Laboratory (Tewksbury, MA, USA). DMEM medium was purchased from Gibco cell culture (Los Angeles, CA, USA). 

***Cell culture and ADR treatment.*** Human breast cancer cell lines MCF7 and MDA-MB-231 were supplied by ATCC (Manassas, VA, USA) and cultured in a medium containing DMEM, 10% fetal calf serum, 2 mM glutamine, 1% penicillin-streptomycin (Gibco, Los Angeles, CA, USA) at 37 °C with 5% CO_2_. Cell viability was assessed using the Vybrant MTT Cell Proliferation Assay (ThermoFisher, Waltham, MA, USA). Senescence-associated over expression and accumulation of β-galactosidase (SA-β-gal) was analyzed using kits obtained from Cell Biolabs (Atlanta, GA, USA). Protein content was determined using the Pierce bicinchoninic acid (BCA) assay obtained from ThermoFisher. For GC–MS based metabolomic profiling experiments, both cell lines were seeded using approximately 500,000 cells onto 10 cm petri dishes in triplicate. Cells were cultured in Dulbecco modified Eagle’s medium (DMEM) containing 0.04 µg/mL ADR for 0, 1, 3, and 5 days, with the media replaced every other day during which time any non-adherent dead cells removed. The cells were then washed twice with ice cold PBS (pH 7.4) for 3 s, and then again with ice cold de-ionized (DI) water for 1 s. Cold extraction solvent (methanol:chloroform, 9:1) was added to quench the cellular metabolism. The cells were detached using a cell scraper and cell suspensions were then transferred into Eppendorf tubes and centrifuged. Supernatants were collected and dried under vacuum using an Eppendorf Concentrator plus (Eppendorf, Hamburg, Germany).

For isotope tracing experiments, the cells were prepared as described above in 10 cm petri dishes and were grown to approximately 90% confluence. Both cell lines were then cultured in medium containing 0.04 µg/mL ADR for 5 days. At the end of ADR treatment, the media were replaced with new medium containing 2 mM [U-^13^C]-glucose for 24 h. Cell metabolite extraction was carried out as described above.

***Sample preparation.*** After drying, cell sample extracts were again dissolved in 200 µL methanol:choloroform (9:1) and centrifuged for 10 min. A 150 µL aliquot of the supernatant was transferred to an Eppendorf tube containing a glass insert and centrifuged under vacuum using an Eppendorf Concentrator plus. Dried extracts were stored at −20 °C until derivatization was performed for GC–MS analysis.

The dried extracts were treated first with 25 μL MeOX (20 mg/mL) reagent at 37 °C for 90 min, followed by derivatization using 75 μL MSTFA with 1% TCMS for 30 min at 37 °C. Finally, 2 μL FAME solution was added to the mixture and vortexed in preparation for GC–MS analysis.

For isotope tracing analysis, the dried extracts were treated first with 30 μL MeOX (20 mg/mL) reagent at 37 °C for 90 min, followed by derivatization with 70 μL MTBSTFA with 1% TBDMCS for 30 min at 70 °C. Finally, 2 μL FAME solution was added to the mixture and vortexed prior to GC–MS analysis.

***Protein content***. Protein concentrations were determined using a BCA assay according to the manufacturer’s instructions. 

***GC–MS analysis.*** All samples were analyzed using an Agilent 7890 GC instrument equipped with a 5975 mass selective detector (MSD), employing an HP-5 ms GC column (30 m length, 0.25 mm i.d., 0.20 µm film thickness). The sample injection (1 μL) was performed in splitless mode at an injection temperature of 250 °C. Helium carrier gas flow was 1.0 mL/min. The ion source temperature was 250 °C. The temperature gradient for GC separation was initially 60 °C for 1 min, then increased from 60 °C to 325 °C at 10 °C/min, where it remained for 10 min. 

***Data preprocessing.*** Retention indices (RIs) were calculated for each sample using AMDIS software (National Institute of Standards and Technology (NIST), Gaithersburg, MD). RI information was subsequently applied to the chromatographic analysis of each sample. The NIST-08 mass spectral and the Agilent Fiehn Metabolomics Retention Time Lock (RTL) Libraries were used to match both mass spectra and RIs to identify metabolites (ΔRI < 2%). For isotope tracing analysis, metabolites were identified based on their RI and mass fragmentation patterns by comparison with metabolite standard compounds. 

Collected GC–MS data were converted from the Agilent ChemStation to MassHunter formats using the Agilent GC/MSD translator. Processing of converted data was performed using Agilent Mass Hunter Quantitative Analysis. First, a batch library was created containing the names of identified compounds, retention times, *m*/*z* values, and their tolerances. Based on the existing library, peak integration and deconvolution were then performed with Mass Hunter using the batch method. A minimum absolute abundance of 1000 counts was used to filter the data. The extracted data were exported from Mass Hunter in “tsv” format, which can be viewed using Microsoft Excel software. 

***Data analysis.*** The extracted data for each cell sample were normalized to protein content and introduced into SIMCA-P software v11.5 (Umetrics, Malmö, Sweden) for PCA to identify outliers and to visualize general data clustering and trends among the observations [35]. The overall metabolic trajectory plots of two cell lines were prepared with Origin 8.5 (OriginLab, Northampton, MA, USA) using the centroids determined from PCA analysis. *p* values comparing metabolites that contribute to discrimination were calculated in Excel using a two-tailed Student’s *t*-test. For isotope tracing analysis, the mean isotope enrichment and isotopotologue distribution of labeled metabolites in the TCA cycle and glycolysis pathway were calculated using IsoCor (http://metasys.insa-toulouse.fr/software/IsoCor/) analysis [36].

## 5. Conclusions

In sum, relatively little is known about the metabolic changes that accompany and maintain cell senescence. Here, we performed metabolomic analyses of ADR-induced senescence in two well-known breast cancer cell lines. Our metabolomics results indicate large and dynamic metabolic changes during ADR-induced cell senescence. Based on metabolic trajectory and univariate analysis, we conclude that the metabolic changes of MCF7 and MDA-MB-231 cells subjected to ADR treatment were characterized by a two-stage process. Moreover, most of the significantly regulated metabolites in the first stage exhibited partial metabolic recovery during the second stage.

**Figure 7:**
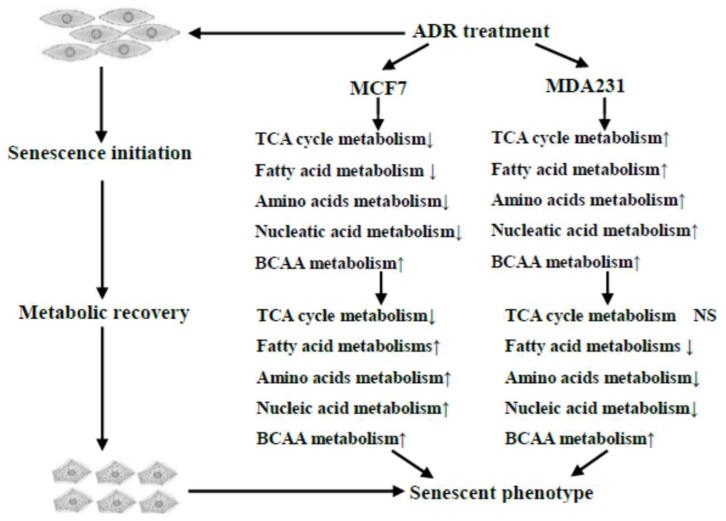
Overview of the dynamic metabolic changes during ADR-induced cell senescence.

Our work demonstrated that ADR treatment induced a number of important metabolic responses in MCF7 and MDA-MB-231 cells, which are illustrated in Figure 7. The metabolic changes observed at earlier treatment times caused by a moderate dosage of ADR included energy, amino acid and lipid metabolisms as well as others. Persistent genotoxic stresses activated cell metabolic recovery as cells went into senescence, a process that might be related to maintaining senescence. Isotope incorporation analysis results suggested a lower glucose flux into the TCA cycle, and glutamine anapleurosis might be a key component to maintain cell senescence in MCF7 (Figure 6c). This pathway potentially can be explored as a therapeutic target to treat senescent cells, with the goal of increasing the vulnerability of cells after mild and repairable genotoxic stress [37].

## Figures and Tables

**Table 1:** Metabolites showing significantly altered levels during cell senescence in the MCF7 cell line and their related metabolic pathways.

MCF7 Cells					
Metabolite	3 Day versus 0 Day		5 Day versus 3 Day		Involved Pathway
	Fold Change	*p*-Value	Fold Change	*p*-Value	
Malic acid	0.42	0.0016	1.85	0.0039	TCA cycle
Fumaric acid	0.58	0.0071	1.69	0.021	
Citric acid	0.21	0.00024	3.69	0.05	
Valine	0.57	0.0019	1.76	0.048	Amino acid metabolism
Leucine	0.56	0.0036	1.73	0.028	
Isoleucine	0.59	0.012	1.66	0.043	
Proline	2.51	0.012	1.39	0.029	
Serine	0.19	0.0061	1.79	0.017	
Aspartic acid	0.34	0.00081	1.81	0.0015	
Glutamine	0.3	0.0084	1.82	0.092	
Lauric acid	0.25	0.0039	2.6	0.0022	Fatty acid metabolism
Palmitic acid	0.28	0.00084	2.57	0.0014	
Linoleic acid	0.37	0.00098	2.02	0.0021	
Heptadecanoic acid	0.067	0.0019	2.73	0.013	
Oleic acid	0.35	0.0042	2.07	0.0086	
Stearic acid	0.5	0.0042	2.63	0.019	
2-Hydrloxyethyl palmitate	0.26	0.0017	2.62	0.0061	
2-Ketobutyric acid, enol	2.12	0.035	1.55	0.093	Branched-chain amino acid (BCAA) metabolism
2-Keto-3-methylvaleric acid	2.94	0.037	2.87	0.061	
Creatinine	0.045	0.016	1.58	0.0028	Nucleic acid metabolism
Glycerol-3-phosphate	0.074	0.0056	1.55	0.03	
Cytindine-5′-monophosphate	0.26	0.049	2.41	0.0096	
Uridine-5-monophosphate	0.0066	0.019	1.45	0.31	
Pantothenic acid	0.34	0.026	1.28	0.069	Others
Cholest-8(14)-en-3-ol	0.031	0.0031	4.79	0.018	

**Table 2:** Metabolites showing significantly altered levels during cell senescence in the MDA-MB-231 cell line and their related metabolic pathways.

MDA-MB-231 Cells					
Metabolite	3 Day versus 0 Day		5 Day versus 3 Day		Involved Pathway
	Fold Change	*p*-Value	Fold Change	*p*-Value	
Malic acid	0.33	0.00045	0.91	0.61	TCA cycle
Fumaric acid	0.44	0.0019	0.88	0.48	
Citric acid	0.2	0.019	2.68	0.1	
Alanine	17.62	0.019	0.47	0.042	Amino acid metabolism
Leucine	2.21	0.00073	0.66	0.0038	
Isoleucine	2.6	0.002	0.59	0.00035	
Proline	3.42	0.01	0.43	0.01	
Glycine	2.2	0.0029	0.47	0.02	
Threonine	2.69	0.0053	0.47	0.0036	
Glutamic acid	1.97	0.0056	0.43	0.00249	
Phenylalanine	4.34	0.014	0.46	0.026	
2-Aminoadipic acid	1.83	0.012	0.42	0.0045	
Tyrosine	2.96	0.0028	0.85	0.021	
L-Tryptophan	5.61	0.029	0.48	0.05	
Heptadecanoic acid	20.42	0.00043	1.55	0.17	Fatty acid metabolism
Oleic acid	1.27	0.04	0.9	0.2	
Stearic acid	0.58	0.0029	1.48	0.19	
2-Keto-3-methylvaleric acid	2.98	0.03	1.12	0.57	BCAA metabolism
Isobutyric acid	4.86	0.023	1.13	0.43	
Glycerol-3-phosphate	21.28	0.0065	1.046	0.81	Nucleic acid metabolism
Creatinine	10.81	0.026	0.75	0.23	
5-Methylthioadenosine	3.7	0.0085	0.65	0.045	
Phosphorylethanolamine	63.71	0.035	0.78	0.52	Others
Pantothenic acid	2.45	0.0075	0.94	0.73	
Scyllo-inositol	0.69	0.023	1.54	0.26	
D-Myo-inositol	1.57	0.0037	0.68	0.13	
Cellobiose	3.7	0.0085	0.65	0.045	
Cholest-8(14)-en-3-ol	19.94	0.00052	0.61	0.044	
